# Editorial: Single-Entity Electrochemistry

**DOI:** 10.3389/fchem.2021.812273

**Published:** 2021-12-09

**Authors:** Wei Ma, Mario A. Alpuche-Aviles, Qianjin Chen, Caleb M. Hill

**Affiliations:** ^1^ Key Laboratory for Advanced Materials and Joint International Research Laboratory of Precision Chemistry and Molecular Engineering, Feringa Nobel Prize Scientist Joint Research Center, Frontiers Science Center for Materiobiology and Dynamic Chemistry, School of Chemistry and Molecular Engineering, East China University of Science and Technology, Shanghai, China; ^2^ Department of Chemistry, University of Nevada, Reno, NV, United States; ^3^ State Key Laboratory for Modification of Chemical Fibers and Polymer Materials, College of Chemistry, Chemical Engineering and Biotechnology, Donghua University, Shanghai, China; ^4^ Department of Chemistry, University of Wyoming, Laramie, WY, United States

**Keywords:** single entity, nanoelectrochemistry, single nanoparticle photoelectrochemistry, nanopore sensing, big data analysis

Single-entity analysis is a holy grail in the analytical chemistry field. When the measurement scale is down to the single entity level, individuals usually exhibit different physical or chemical performance from the ensemble or bulk state. However, traditional measurements all focus on the bulk responses, which neglect the structure heterogeneities and obscure the intrinsic performance of individual entities (Baker, 2018). Recently, single-entity electrochemistry provides a powerful method in high-throughput detecting and characterizing individual entities, that is, nanoparticle, virus, nanobubble, even molecule and atom. Due to low cost, little sample consumption, and rapid and sensitive measurement, the fundamental research and practical application of single-entity electrochemistry has rapidly developed in recent decades (Crooks, 2016). Considering the transient nature and the ultralow amplitude of individual entities, a critical barrier of single-entity electrochemical measurement is obtaining the weak signals among the noise (Ma, et al., 2017). On the one hand, reducing the electrode size into a micro- or nanoscale is an effective way to lower the background noise and match the single analytes. On the other hand, the rapid development of low-noise electrochemical instruments allows accurately measuring the electrochemical signal of an individual with high temporal and current resolution.

As an emerging and convenient electrochemical method, stochastic collision electrochemistry has been used to investigate the intrinsic properties of individual entities, including size, shape, concentration, aggregation degree, and catalytic performance, during their stochastic collision at an ultramicroelectrode (Sun, et al., 2019). Thanks to the large data volume, the unavoidable background noise, and the filtering effect in single-entity electrochemical experiments, the accurate analysis of the current signal based on big data analysis is of great significance to efficiently access accurate information of individual events. In this research topic, Zhao et al. proposed a spike detection algorithm for automatically processing the data from the direct oxidation of silver nanoparticles in single-nanoparticle collision experiments, including baseline extraction, spike identification, and spike area integration.

The development of nanoelectrode offers an important advantage in spatial resolution for scanning electrochemical microscopy, scanning electrochemical cell microscopy, scanning ion conductance microscopy, etc (Kai, et al., 2018; Wahab, et al., 2020). The nanoscale imaging ability due to the high spatial resolution enables single-entity analysis to be more useful. Moreover, the state and expansion of the electrode, such as nanopore, nanopipette, nanoelectrode, microphase-separated block copolymer thin film, and others, have attracted considerable attention. Nanopore sensing is a powerful tool in single-entity electrochemical measurement due to the high sensitivity and versatility of this technique. It has been widely applied in detecting metal ions, small molecules, nucleotides and proteins in the past several decades, especially in DNA sequencing (Deamer, et al., 2016). In this Research Topic, the mini-review by Yang et al. focuses on the recent progress in the modification and characterization of TEM-fabricated nanopores. Moreover, some key applications of these nanopores are highlighted in nucleic acids, protein, and nanoparticle detection. Additionally, they discuss the future of computer simulations in DNA and protein sequencing strategies based on nanopore detection technology.

Interest in single-entity photoelectrochemical experiments arises from the need to understand the implications of materials under illumination at the nanometer scale. These photoelectrochemical processes are more challenging than the electrocatalytic reactions that operate in the absence of light. Furthermore, in the presence of light, materials’ behavior depends on several additional experimental features, for example, illumination power. Because of these different practical issues, progress on single-entity photoelectrochemistry has been slower than in other electrochemical areas. This topic includes two contributions that address the single-entity photoelectrochemistry. Mathuri et al. review the current literature of single-entity studies of semiconducting materials in the broader context of single-entity electrochemistry. Subedi et al. demonstrate the detection of colloidal CdSe and CdSe/ZnS QDs. The authors have observed the stepwise photocurrent vs. time response, which people in the field now take as the “signature” of the adsorption/desorption of single entities. Therefore, the materials CdSe and CdSe/ZnS irreversibly adsorb to the Pt ultramicroelectrode. However, the observed photocurrent is relatively large, > 1 pA, which indicates that the entities adsorbing to the UME are aggregates in this work. These results were consistent with lower collision frequencies than the diffusion-limited value expected for single QDs/UME interactions. Also, dynamic light scattering and scanning electron microscopy studies are consistent with the detection of agglomerates.

Nowadays, single-entity electrochemical measurement coupled with complementary techniques such as optical microscopy, plasmonic and Raman scattering techniques makes it possible to identify the full picture of single-NP electrochemical behaviors with sufficient information (Wang, et al., 2016). Two of the original research articles included here focus on the electrochemical behaviors at the individual nanoparticle level by combining other imaging techniques. Garcia et al. report on using a plasmonic electrochemical microscope to study the electrocatalytic reduction of Prussian blue nanoparticles for hydrogen peroxide at the individual nanoparticle level. On the other hand, Cashen et al. explore an optically detected electrochemical approach that uses a conventional bright-field optical microscope to acquire single-particle electrochemical data and *ex situ* TEM characterization of single nanoparticles in a one-to-one fashion. The results are significant for correlated electrochemical/TEM imaging studies that aim to reveal structure–property relationships using single particle–level imaging and ensemble-level electrochemistry.

Looking at current developments, we may foresee that single-entity electrochemistry is not only a commercial success in analytical applications but also a diverse tool to answer fundamental questions in chemistry, biology, and physics. The articles on this research topic show how the single-entity electrochemical application expands by combining electrochemical measurement with *in situ* and *in operando* imaging techniques. These measurements will be helpful to further understand the intrinsic properties of individual entities to critical electron transfer processes occurring at the micro-/nano-interface.
